# Production and characterization of novel monoclonal antibodies against pathological human TDP-43 proteins

**DOI:** 10.1093/jnen/nlae042

**Published:** 2024-05-10

**Authors:** Xiaojing Zheng, Mengtian Wang, Qiongyan He, Shuyu Chen, Dilihumaer Simayi, Xia Ma, Junli Zhao, Xiaohong Sun, Peiyan Yang, Qinwen Mao, Haibin Xia

**Affiliations:** Laboratory of Gene Therapy, Department of Biochemistry, College of Life Sciences, Shaanxi Normal University, Shaanxi, P.R. China; Laboratory of Gene Therapy, Department of Biochemistry, College of Life Sciences, Shaanxi Normal University, Shaanxi, P.R. China; Laboratory of Gene Therapy, Department of Biochemistry, College of Life Sciences, Shaanxi Normal University, Shaanxi, P.R. China; Laboratory of Gene Therapy, Department of Biochemistry, College of Life Sciences, Shaanxi Normal University, Shaanxi, P.R. China; Laboratory of Gene Therapy, Department of Biochemistry, College of Life Sciences, Shaanxi Normal University, Shaanxi, P.R. China; Laboratory of Gene Therapy, Department of Biochemistry, College of Life Sciences, Shaanxi Normal University, Shaanxi, P.R. China; Laboratory of Gene Therapy, Department of Biochemistry, College of Life Sciences, Shaanxi Normal University, Shaanxi, P.R. China; Laboratory of Gene Therapy, Department of Biochemistry, College of Life Sciences, Shaanxi Normal University, Shaanxi, P.R. China; Laboratory of Gene Therapy, Department of Biochemistry, College of Life Sciences, Shaanxi Normal University, Shaanxi, P.R. China; Department of Pathology, University of Utah, Salt Lake City, Utah, USA; Laboratory of Gene Therapy, Department of Biochemistry, College of Life Sciences, Shaanxi Normal University, Shaanxi, P.R. China

**Keywords:** Alzheimer disease, Frontotemporal lobar degeneration, Phosphorylation-dependent antibody, Phosphorylation-independent antibody, Proteinopathy, TDP-43

## Abstract

The RNA/DNA-binding protein TDP-43 plays a pivotal role in the ubiquitinated inclusions characteristic of TDP-43 proteinopathies, including most cases of frontotemporal lobar degeneration (FTLD-TDP) and Alzheimer disease (AD). To understand the mechanisms of pathological TDP-43 processing and identify potential biomarkers, we generated novel phosphorylation-independent monoclonal antibodies (MAbs) using bacteria-expressed human full-length recombinant TDP-43. Remarkably, we identified a distinctive MAb, No. 9, targeting an epitope in amino acid (aa) region 311–360 of the C-terminus. This antibody showed preferential reactivity for pathological TDP-43 inclusions, with only mild reactivity for normal nuclear TDP-43. MAb No. 9 revealed more pathology in FTLD-TDP type A and type B brains and in AD brains compared to the commercial p409/410 MAb. Using synthetic phosphorylated peptides, we also obtained MAbs targeting the p409/410 epitope. Interestingly, MAb No. 14 was found to reveal additional pathology in AD compared to the commercial p409/410 MAb, specifically, TDP-43-immunopositive deposits with amyloid plaques in AD brains. These unique immunopositivities observed with MAbs No. 9 and No. 14 are likely attributed to their conformation-dependent binding to TDP-43 inclusions. We expect that this novel set of MAbs will prove valuable as tools for future patient-oriented investigations into TDP-43 proteinopathies.

## INTRODUCTION

Transactive response DNA/RNA-binding protein of 43 kDa (TDP-43) is the primary component of the ubiquitin-positive, tau-negative inclusions found in frontotemporal lobar degeneration with TDP-43 pathology (FTLD-TDP) (1–3). TDP-43 is composed of 414 amino acids and functions as a heterogeneous nuclear ribonucleoprotein (4). It contains several well-studied domains, including 2 highly conserved RNA-recognition motifs and a glycine-rich C-terminal domain (5, 6). Under normal conditions, TDP-43 mainly localizes to the nucleus, where it regulates gene transcription and mRNA splicing (7–9). In neurodegenerative conditions, TDP-43 changes conformation, misfolds, and accumulates in intranuclear and cytoplasmic inclusions (1, 10–12). These inclusions cause loss of nuclear TDP-43 function and are themselves toxic (13, 14). TDP-43 inclusions are predominantly composed of pathologic TDP-43 proteins: mainly 45 kDa phosphorylated full-length TDP-43 and 25 kDa C-terminal fragments (1, 10). Notably, both the full-length and C-terminal fragments of TDP-43 are phosphatase-sensitive, indicating disease-associated hyperphosphorylation. The phosphorylation sites of TDP-43 are mostly located in the glycine-rich C-terminal domain of the protein (15, 16).

Patients with FTLD-TDP display clinical, pathological, and genetic heterogeneity. Clinically, FTLD-TDP can present with a wide range of phenotypes, encompassing behavioral/executive dysfunction with or without motor neuron disease, as well as language dysfunction (17). Pathologically, the brain regions affected by FTLD-TDP demonstrate a prevalence of cytoplasmic or neuritic, insoluble, and phosphorylated TDP-43 inclusions within their neurons and glia (1, 18). Despite these common features, there exists significant pathological heterogeneity in the distribution and morphology of TDP-43 inclusions within TDP-43 proteinopathies. Studies have identified at least 5 distinct histopathologic subtypes—A to E—of TDP-43 pathology in FTLD-TDP (19–22). These studies relied on classifying cases via immunohistochemistry using type-specific antibodies. The most commonly used antibody is the one that binds to the phosphorylated serine at aa409/410 (p409/410) of the C-terminus of TDP-43; it is believed that this antibody can detect pathologic forms of TDP-43. Since 2006, this histopathologic classification has been widely used to reveal the association between FTLD-TDP pathology and clinical phenotypes. Yet, there has been major controversy regarding a causal link between TDP-43 pathology and neurodegeneration. For example, we observed that the density of TDP-43-positive inclusions revealed by the p409/410 monoclonal antibody (MAb) may show no or an inverse correlation with local neurodegeneration (23), possibly because the p409/410 MAb is not entirely specific for pathologic TDP-43 (24).

The heterogeneity of FTLD-TDP pathology is reflected in TDP-43’s complex molecular composition and toxic properties. TDP-43 inclusions likely represent a mixture of toxic and nontoxic TDP-43 species (25). Laferrière et al showed that the molecular properties of TDP-43 inclusions are correlated with specific neuropathological subtypes. Using SarkoSpin, a method for biochemically isolating pathologic TDP-43, they showed that the extracted TDP-43 assemblies exhibit cytotoxicity that reflects the disease duration of the respective subtype (25). These observations indicate that toxic TDP-43 species might be a link between a defined TDP-43 pathology and a specific clinical phenotype. Biomarkers, preferably antibodies, for the new toxic TDP-43 species need to be discovered to confidently link pathology to clinical phenotype, thus improving our ability to diagnose patients.

Soon after the initial characterization of TDP-43-immunoreactive inclusions in FTLD, these inclusions were also identified in 25%–60% of pathologically confirmed Alzheimer disease (AD) cases (26, 27). TDP-43-positive inclusions in AD appear first in the amygdala, followed by the entorhinal cortex and hippocampus, occipitotemporal cortex, insular and inferior temporal cortex, brainstem, frontal cortex, and basal ganglia (26–31); this a scheme that has been independently validated (32). TDP-43 inclusions are heterogeneous in AD. Josephs et al classified TDP-43-positive inclusions into TDP-43 type α and type β (33). Type α inclusions are “typical” TDP-43-immunoreactive inclusions; type β inclusions are those adjacent to/associated with neurofibrillary tangles in the same neuron. Limbic-predominant age-related TDP-43 encephalopathy neuropathological change (LATE-NC) has recently been proposed as a term to describe the presence of TDP-43-positive inclusions in AD, other dementias, and older adults (34, 35). Although LATE-NC research has become a hot topic in the last few years, the term LATE-NC has been questioned for its “novelty and nosology” due to unclear biochemical differences underlying TDP-43-immunoreactive inclusions in FTLD-TDP vs non-FTLD-TDP disorders (36, 37). Similarly, additional antibodies targeting potential new TDP-43 species are required to enhance our understanding of LATE-NC.

In this study, novel MAbs against TDP-43 were produced and characterized using bacteria-expressed human full-length recombinant TDP-43 protein and synthetic peptides containing the p409/410 epitope. We obtained the novel phosphorylation-independent MAb No. 9 and phosphorylation-dependent MAb No. 14. MAb No. 9 revealed more pathology in FTLD-TDP type A and type B brains, and LATE-NC brains than the p409/410 MAb. Additionally, MAb No. 14 was able to reveal new pathology in LATE-NC brains compared to the p409/410 MAb.

## MATERIALS AND METHODS

### Plasmid construction of full-length human TDP-43 and truncation mutants

**Figure 1.**
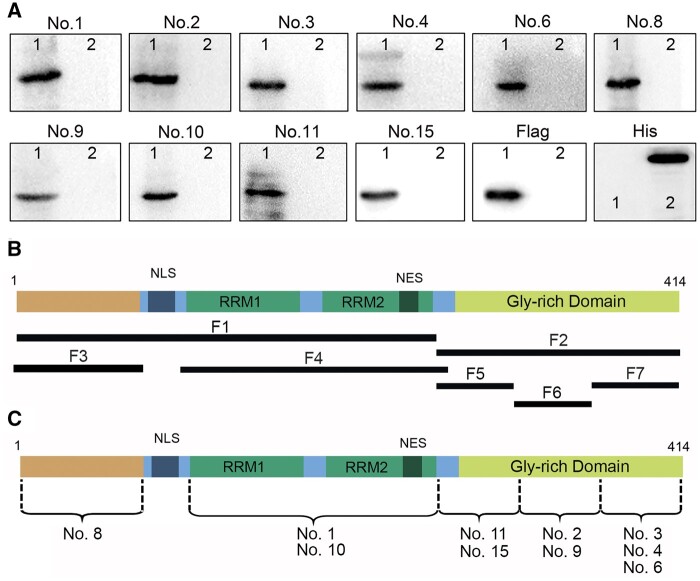
Phosphorylation-independent MAb production and epitope mapping. **(A)** Reactivity of MAbs with TDP-43-Flag recombinant protein of about 46 kDa (lane 1) overexpressed in HEK 293 cells by Western blot. Anti-Flag MAb (Flag) was used as the positive control antibody. Leucine-rich repeat-containing protein 15 (LRRC15)-His fusion protein (lane 2) served as the negative control, which was revealed by anti-His MAb (HIS). **(B)** Schematic representation of the TDP-43 fragments employed for epitope mapping. **(C)** Mapping of TDP-43 domains with epitopes recognized by the 10 generated MAbs. This mapping was performed across the entire length of TDP-43 using immunoblot and indirect peptide ELISA. RRM, RNA-recognition motif; NLS, bipartite nuclear localization signal; NES, nuclear export signal.

Full-length human TDP-43 (hTDP-43) cDNA was amplified using the MegaMan Human Transcriptome Library (Agilent-Stratagene, Santa Clara, CA) as a template through nested PCR. Following gel purification, the amplified products were cloned into the pGEM-T easy vector (Promega, Madison, WI). Positive clones were confirmed through restriction enzyme digestion and sequencing, resulting in a plasmid named pGEMT-hTDP-43. The truncation mutants of hTDP43 were obtained using PCR based on the template of pGEMT-hTDP-43. The resultant plasmids were called pGEMT-hTDP-43 truncation mutants. The primer sets are detailed in Table 1, and the positions of the truncation mutants in the TDP-43 gene are shown in Figure 1. Subsequently, the full-length hTDP-43 or hTDP-43 truncation mutants were inserted into the pRSET-B/His6 vector and pcDNA3.1/Flag (Invitrogen, Carlsbad, CA), respectively. The obtained plasmids were named pRSET-hTDP-43, pRSET-hTDP-43 truncation mutants, pcDNA3.1/hTDP43-Flag, or pcDNA3.1/hTDP43 truncation mutants-Flag.

**Table 1.** Primers for full-length human TDP-43 and truncation mutants

Primers	Sequences
Human TDP-43 *Cla*I forward	AATCGATATGTCTGAATATATTCGGGTAACCG
Human TDP-43 *Xba*I reverse	ATCTAGATTACATTCCCCAGCCAGAAGACTTA
Human TDP-43 no stop *Xba*I back	ATCTAGACATTCCCCAGCCAGAAGACT
Human TDP-43 aa1-262 *Xba*I reverse	ATCTAGATTATTCGGCATTGGATATATGAA
Human TDP-43 AA262-414 *Cla*I forward	AATCGATATGCCTAAGCACAATAGCAATAG
Human TDP-43 aa1-77 *Bgl*II forward	AAGATCTATGTCTGAATATATTCGGGT
Human TDP-43 aa1-77 *Bstb*I reverse	ATTCGAATTAATAGTTGACAACATACAC
Human TDP-43 aa311-360 *Bgl*II forward	AAGATCTATGAACTTTGGTGCGTTC
Human TDP-43 aa311-360 *Bstb*I reverse	ATTCGAATTACTGCATGTTGCCTTGGTT
Human TDP-43 aa102-267 *Cla*I forward	AATCGATATGAAAACATCCGATTTAATAGT
Human TDP-43 aa102-267 *Xba*I reverse	ATCTAGATTAATTGCTATTGTGCTTAGGTT
Human TDP43 aa263 *Bgl*II forward	AAGATCTAAGCACAATAGCAATAGA
Human TDP43 aa263 *Bgl*II forward	ATTCGAATTACCCACCACCCATATTACT
Human TDP43 aa361 *Bgl*II forward	AAGATCTAGGGAGCCAAACCAGGCC
Human TDP43 *Bstb*I reverse stop	ATTCGAACTAATTCCCCAGCCAGAAGA

### Expression of full-length hTDP-43 and truncation mutants in *Escherichia coli* BL21

The pRSET-hTDP-43 and pRSET-hTDP-43 truncation mutants were transformed into *E. coli* BL21 (DE3) for the prokaryotic protein expression according to the methods described in our previous study (38). Then, purified hTDP-43 proteins and hTDP-43 truncation mutants were obtained and stored at −80°C and used for subsequent experiments.

### MAb production and purification

Six 5-week-old female BALB/c mice (procured from the Animal Center of Shaanxi Normal University, Xi’an, China) were immunized 3 times at 3-week intervals with approximately 40 µg of purified hTDP-43-6His or 4 times at 2-week intervals with 80 µg of KLH- or OVA-conjugated TDP-43 peptide containing p409/410 [MDSKS(p)S(p)GWGM] (Thermo Fisher Scientific, Rockford, IL). Animal care and all procedures performed here were approved by the Shaanxi Normal University Animal Care and Use Committee. The purified hTDP-43-6His or KLH- or OVA-conjugated TDP-43 p409/410 peptide was emulsified in complete Freund’s adjuvant (Sigma, Milwaukee, WI) for the first injection and then in incomplete Freund’s adjuvant (Sigma) for the second and third injections. The first and second injections were administered subcutaneously, and the third injection was intraperitoneal. Blood samples were collected from the mice 15 days after the last immunization to determine antibody titers via indirect enzyme-linked immunosorbent assay (ELISA). Pre-immunized sera served as the negative control. Sera from all 6 mice exhibited a titer of at least 1:32 000 in the ELISA.

SP2/0 myeloma cells were fused with spleen cells isolated from immunized BALB/c mice using standard methodology, as detailed in our previous study (38). The positive hybridoma cells secreting MAbs against hTDP-43 or p409/410 were obtained after screening with the hTDP-43 peptide, bovine serum albumin (BSA)-conjugated p409/410 peptide or BSA-conjugated 409/410 peptide. All MAbs were then purified for subsequent experiments using the affinity chromatography method.

### ELISA and MAb subclass determination

For indirect ELISA, 0.5–1 µg of protein was coated. For sandwich ELISA, 1 µg of capturing antibody was coated, followed by the application of a 1:1000 dilution of biotin-labeled detecting antibody; subsequently, 1:3000 Avidin-HRP was added. MAb subclass was determined using the Mouse MAb Isotyping Kit (Sigma). Coating was carried out in 0.05 M of bicarbonate buffer (pH 9.6) overnight at 4°C. Standard methodology was employed, as detailed in our previous study (38).

### Cell culture and transfection

Two cell lines, SH-SY5Y (a human neuroblastoma cell line) and HEK 293 (a human embryonic kidney cell line), were sourced from the American Type Culture Collection (ATCC, Manassas, VA). All cells were cultured in Dulbecco’s modified Eagle’s medium (Gibco, Grand Island, NY) supplemented with 10% (vol/vol) fetal bovine serum (Gibco, Grand Island, NY), 1% penicillin-streptomycin, and 1% L-glutamate. The cell lines were maintained in a humidified chamber with 5% CO_2_ at 37°C. HEK 293 cells were overexpressed with hTDP-43, hTDP-43-Flag, and hTDP-43 truncation mutants by transfecting the corresponding plasmids. HEK 293 cells were transfected using the standard calcium phosphate method.

### In vitro phosphorylation of TDP-43 protein

For in vitro phosphorylation, 2 µg of recombinant TDP-43 (ACROBiosystems, Newark, DE) were incubated with 1 µg of Casein Kinase 1 (CK1) alpha 1 (Sigma, St Louis, MO) in 1× kinase buffer (25 mM Tris-HCl [pH 7.5], 5 mM beta-glycerophosphate, 2 mM dithiothreitol, 0.1 mM Na_3_VO_4_, 10 mM MgCl_2_) containing 200 mM ATP at 30°C for 12–18 hours.

### Human postmortem cases

This project took advantage of brain samples from the Gift to Life brain bank in the Department of Pathology at the University of Utah. The average postmortem interval of the brains in this brain bank is 13.5 hours. Brains with postmortem intervals greater than 24 hours were excluded from this study, as were brains from patients with a significant medical illness that would interfere with pathologic diagnosis (e.g. brain tumor). Paraformaldehyde-fixed, paraffin-embedded human brain samples from 12 cases were obtained. Demographic and neuropathologic data for these cases are presented in Table 2. The cases were subdivided into 4 groups: normal control (CON), AD, AD with limbic-predominant age-related TDP-43 encephalopathy neuropathological change (LATE-NC), FTLD-TDP (including type A, type B, and type C cases). The immunostaining and frozen tissue extraction steps were both performed on tissue from these cases. For the immunostaining study, the frontal cortical sections were used to evaluate the anti-TDP-43 MAbs in the FTLD-TDP cases. Sections of the frontal cortex, hippocampus, and amygdala were used to evaluate the anti-TDP-43 MAbs in the AD with LATE-NC cases. Pathologic characterization was conducted by board-certified neuropathologists following consensus criteria (2, 39–41). Informed consent was obtained for all studies.

**Table 2.** Sample demographics

Case	Neuropathologic diagnosis	Gender M/F	Age at death (years)	Disease duration (years)	Mutation	Clinical diagnosis	ADNC score (A, B, C)	LATE-NC stage
1	CON	M	72	NA	None	NA	0, 1, 0	NA
2	CON	F	69	NA	None	NA	1, 1, 0	NA
3	CON	F	79	NA	None	NA	0, 0, 0	NA
4	AD	M	77	11	None	Probable AD	3, 3, 3	NA
5	AD	F	69	9	None	Probable AD	3, 3, 3	NA
6	AD	F	66	10	None	Probable AD	3, 3, 3	NA
7	AD with LATE-NC	M	78	12	None	Probable AD	3, 3, 3	2
8	AD with LATE-NC	F	69	11	None	Probable AD	3, 3, 3	2
9	AD with LATE-NC	F	82	9	None	Probable AD	3, 3, 3	2
10	FTLD-TDP type A	M	72	7	None	FTD	1, 1, 1	NA
11	FTLD-TDP type A	F	69	6	None	FTD	0, 1, 0	NA
12	FTLD-TDP type A	F	66	9	None	FTD	1, 0, 0	NA
13	FTLD-TDP type B	M	64	3	None	FTD	0, 0, 0	NA
14	FTLD-TDP type C	M	72	11	None	FTD	0, 1, 0	NA

CON, normal control; AD, Alzheimer’s disease; LATE-NC, Limbic-predominant age-related TDP-43 encephalopathy neuropathological change; FTLD-TDP type A, frontotemporal lobar degeneration with TDP-43 pathology type A; ADNC, Alzheimer’s disease neuropathologic change; FTD, frontotemporal dementia; NA, not applicable.

### Fractionation of brain extracts

Fractionation of brain extracts (10, 22, 42, 43) involved frozen temporal or frontal cortex samples (0.5 g) from AD, FTLD-TDP type A, and normal control cases. The samples were homogenized in 10 volumes (5 ml) of buffer A (10 mM Tris-HCl [pH 7.5], 1 mM EGTA, 10% sucrose, and 0.8 M NaCl). After adding another 5 ml of buffer A containing 2% Triton X-100, the homogenate was incubated for 30 minutes at 37°C and spun at 100 000 × *g* for 30 minutes at 4°C. The resulting pellet was homogenized in 5 volumes of buffer A, followed by a 30-minute incubation at 37°C with 1% sarkosyl. The homogenate was then spun at 100 000 × *g* for 30 minutes at room temperature. The sarkosyl-insoluble pellet was homogenized in 4 volumes of buffer A containing 1% CHAPS and spun at 100 000 × *g* for 20 minutes at room temperature. The pellet was sonicated in 0.6 volumes of 7 M guanidine hydrochloride, followed by overnight dialysis at 4°C against 30 mM Tris-HCl (pH 7.5).

### Western blot

Samples containing recombinant proteins hTDP-43-Flag, hTDP-43, and hTDP-43 truncation mutants overexpressed in HEK 293 cells, TDP-43 peptides, SH-SY5Y cells, or patients’ brain tissue were separated by 10% SDS-PAGE or 12% SDS-PAGE. Eukaryotic-expressed hTDP-43-Flag and hTDP-43 were employed to assess the specificity of antibodies, with hTDP-43 truncation mutants used for epitope mapping of antibodies. Patient brain tissues were used to pathologically characterize the antibodies. The proteins were subsequently electrotransferred onto methanol-pretreated polyvinylidene difluoride (PVDF) membranes (Millipore, Temecula, CA). For immunoblotting, the PVDF membranes were incubated with anti-TDP-43 MAbs overnight at 4°C. After washing, the membranes were detected using HRP-conjugated goat anti-mouse IgG antibody (1:10 000 dilution, Thermo Fisher Scientific), followed by enhanced chemiluminescence Western blot detection reagents (Thermo Fisher Scientific), as per the manufacturer’s protocol.

### Immunofluorescence and immunohistochemistry

For the immunofluorescent detection of endogenous TDP-43 in SH-SY5Y cells or in mouse brains, permeabilized cells or sections were incubated with purified anti-TDP-43 MAbs (1:4000 v/v) overnight at 4°C. After washing 3 times with 1 × PBS, the cells or sections were incubated with FITC-conjugated goat anti-mouse IgG (1:500 v/v, Abcam, Cambridge, MA) for 1 hour at room temperature. Subsequently, the cells or sections were washed with 1×PBS, and sections were mounted in Vectashield-hard set medium containing DAPI (Vector Laboratories, Burlingame, CA). Images were acquired using an Olympus BX53 microscope (Olympus, Tokyo, Japan) with an Olympus DP74 camera and cellSens Dimension software. Images were obtained in either the green or blue channel.

To detect TDP-43 pathology in human brains, 5-µm serial sections of human FTLD-TDP, AD, and normal control brains were used. TDP-43 immunohistochemistry with anti-TDP-43 MAbs was performed, following the staining procedure detailed in our previous study (44). Specifically, the paraffin sections were first deparaffinized and hydrated through xylenes and graded alcohol series. Then, quenching of endogenous peroxidase activity was achieved by incubating the slides in freshly prepared 3% hydrogen peroxide diluted in methanol for 10 minutes at room temperature. Antigen retrieval was then performed in a Decloaking Chamber (Biocare Medical, Pacheco, CA) for 15 minutes using a citrate buffer, pH 6.0. Following washes in distilled water, sections were blocked in 5% goat serum at room temperature for 1 hour. Sections were then incubated in primary antibodies including the purified anti-TDP-43 MAbs (1:4000, v/v), and the phosphorylated TDP-43 (pTDP-43) antibody (pS409/410, 1:4000, v/v, mouse monoclonal, Cosmo Bio Co., LTD, Carlsbad, CA) served as the control antibody. Biotinylated secondary antibodies (Dako, Carpinteria, CA) were amplified using avidin-biotin substrate (ABC solution, Dako), followed by color development in DAB chromogen (K4007, Dako). Images were obtained using an Olympus BX53 microscope with an Olympus DP74 camera, and cellSens Dimension software was used for brightness and contrast adjustment.

The evaluation and semiquantification of non-pathological nuclear TDP-43 and pathological TDP-43 inclusions, including neuronal cytoplasmic inclusions (NCIs), dystrophic neurites (DNs), neuronal intranuclear inclusions (NIIs), and white-matter glial cell inclusions (GCIs), were conducted in frontal cortex samples from patients’ brains. Two investigators, blinded to diagnosis, assessed the extent of the TDP-43 pathology on a semiquantitative scale (0 = none, + = mild, ++ = moderate, or +++ = severe).

Double-label immunofluorescence was performed on selected sections and cases using pTDP-43 polyclonal antibody (PAb) (pS409/410, 1:000, rabbit polyclonal, Cosmo Bio Co., LTD) and MAb No. 9 antiserum. To investigate possible co-localization of MAb No. 9 or No. 14 with a subset of neurofibrillary tangles, double-label immunofluorescence was performed on AD with LATE-NC cases using MAb No. 9 and a rabbit monoclonal anti-tau (Ser202, Thr205) antibody (AT8) (Abcam). To investigate possible co-localization of MAb No. 14 with a subset of plaques, double-label immunofluorescence was performed on AD cases using MAb No. 14 and a rabbit monoclonal anti-beta amyloid (mOC64) (Abcam). After the primary antibodies were incubated overnight at 4°C in a humidified chamber, sections were washed in 0.1 M Tris buffer and then incubated with species-specific secondary antibodies for 2 hours at room temperature (Alexa Fluor 488 conjugated anti-rabbit and cy3 conjugated anti-mouse secondary antibodies, 1:500, Abcam). Sections were mounted in Vectashield-hard set medium containing DAPI (Vector Laboratories). Similarly, the images were acquired using an Olympus BX53 microscope (Olympus) with an Olympus DP74 camera and the cellSens Dimension software. Images were obtained in either the red or green channel. Colocalization was evaluated using the JACoP plugin of the ImageJ software package (NIH, https://imagej.nih.gov/ij/). Threshold-based object recognition was applied first, then the Pearson’s correlation coefficient was calculated. Mean values between zero and one were obtained, with one representing a complete color-mix of the 2 channels (complete yellow corresponding to red and green colocalization), and zero representing the presence of only one marker (either green or red) in the respective region of interest. Pearson’s coefficient values equal to or above 0.5 were considered significant.

### Absorption tests

Absorption tests were used to further confirm the specificity of the antibodies. No. 14 was preabsorbed with beta amyloid-peptide (1–42), TDP-43 p409/410 peptides [MDSKS(p)S(p)GWGM], or TDP-43 409/410 peptides (MDSKSSGW GM) (1 µg/mL) (Thermo Fisher Scientific) prior to applying the antibody to the brain tissue for immunostaining or to the membrane for Western blot.

### Screening of antibody pairs for HRP-based double antibody sandwich ELISA

A sandwich ELISA, employed to identify the optimal antibody pairs, was conducted as previously described (45, 46). The 4 high-affinity, purified MAbs (No. 3, No. 4, No. 6, and No. 9) were biotin-labeled using an EZ-Link Sulfo-NHS-LC-Biotinylation Kit (Thermo Fisher Scientific). Then, a total of 1 µg of each unlabeled MAb (No. 3, No. 4, No. 6, and No. 9) in 100 µL coating buffer (0.05 M carbonate/bicarbonate buffer, pH 9.6) was individually added to each well and incubated overnight at 4°C. After washing with 0.01 M of PBS containing 0.1% Tween-20 (PBST), the wells were blocked with 200 µL of PBST containing 10% fetal bovine serum at 37°C for 2 hours. Following washing, 100 µL of diluted, purified bacteria-expressed TDP-43 was added to the wells and incubated at 37°C for 1 hour. Biotin-labeled antibodies (1:1000, v/v) were added to the wells, followed by incubation with commercial avidin-HRP (1:3000, v/v, Thermo Fisher Scientific). After washing, 100 µL of 0.1 mg/mL TMB containing 0.03% H_2_O_2_ (v/v) substrate solution was added to each well, and the plates were incubated at 37°C for 10 minutes. Finally, 50 µL of 2M H_2_SO_4_ was added to stop the reaction, and the OD values were read at 450 nm using an automated microplate reader. The optimal antibody pairs were selected based on the light absorbance of each well in the plates.

## RESULTS

### Production and epitope mapping of phosphorylation-independent MAbs

MAbs were generated against bacteria-expressed full-length recombinant hTDP-43 protein. Over 5000 MAb clones from 5 hybridoma fusion procedures were screened through indirect ELISA. Following the primary screen, 10 high-affinity MAbs, designated as MAb No. 1–4, No. 6, No. 8–11, and No. 15, were produced. These MAbs are phosphorylation-independent, as they were raised against a bacteria-expressed, unphosphorylated TDP-43 protein. All 10 antibodies specifically reacted with TDP-43-Flag protein overexpressed in HEK 293 cells in Western blot analyses (Fig. 1A, Supplementary Data Fig. S1). Subsequently, these antibodies underwent epitope mapping (38), with different TDP-43 fragments designed based on previously reported functional domains (47) (Fig. 1B). MAb No. 8 recognized the N-terminal domain (aa1-77). MAbs No. 1 and No. 10 targeted the tandem RNA-recognition motif (RRM)1 and RRM2 region (aa102-269). MAbs No. 11 and No. 15 reacted with aa262-310 in the C-terminal area. No. 2 and No. 9 recognized aa311-360 in the C-terminal area. MAbs No. 3, No. 4, and No. 6 reacted with aa361-414 of the C-terminal area (Fig. 1C).

**Figure 2.**
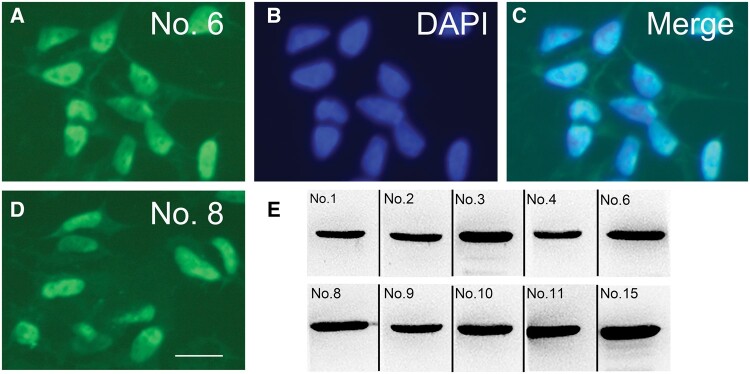
Immunofluorescence staining and Western blot analysis of endogenous TDP-43 in SH-SY5Y cells. **(A–D)** Representative images of immunofluorescence staining using MAbs. MAb No. 6 (A–C) and No. 8 (D) are illustrative of the 10 MAbs detecting normal TDP-43 (green) in the nuclei (blue) of SH-SY5Y cells. Scale bar: 50 µm. (**E**) Western blot employing MAbs on whole-cell lysates of SH-SY5Y cells. All MAbs exhibited strong immunoreactivity with a 43-kDa band.

All antibodies successfully detected endogenous TDP-43 in SH-SY5Y cells by immunofluorescence (Fig. 2A–D). Additionally, all antibodies demonstrated the ability to detect endogenous TDP-43 in SH-SY5Y cells by immunoblotting (Fig. 2E, Supplementary Data Fig. S2). Subsequent evaluations, including by immunoprecipitation, immunohistochemistry, and sandwich ELISA, confirmed the efficacy of all MAbs, as detailed in Table 3.

**Table 3.** Summary of the characterization of the new TDP-43 MAbs

MAb	Isotype	Epitope (aa)	IP	IB	IHC	ICC	ELISA capture	ELISA detect	Human preference
No. 1	IgG2a	102–269	ND	+	−	+/−	ND	ND	−
No. 2	IgG2a	311–360	ND	+	+/−	+/−	ND	ND	−
No. 3	IgG2a	361–414	+	+	+	+	+	+	–
No. 4	IgG2a	361–414	+	+	+	+	+	+	−
No. 6	IgG1	361–414	+	+	+	+	+	+	−
No. 8	IgG2a	1–77	+	+	+	+	+	+	−
No. 9	IgG2a	311–360	+	+	+	+	+	+	+
No. 10	IgG1	102–269	ND	+	+	+	ND	ND	−
No. 11	IgG2a	262–310	ND	+	+	+	ND	ND	−
No. 15	IgG2a	262–310	ND	+	+	+	ND	ND	−
No. 14	IgG1	p409/410	+	+	+	+	+	+	+

ND, not done; IP, immunoprecipitation; IB, immunoblotting; IHC, immunohistochemistry; ICC, immunocytochemistry; ELISA, enzyme-linked immunosorbent assay; aa, amino acid; MAb, monoclonal antibody; +, positive; −, negative.

**Figure 3.**
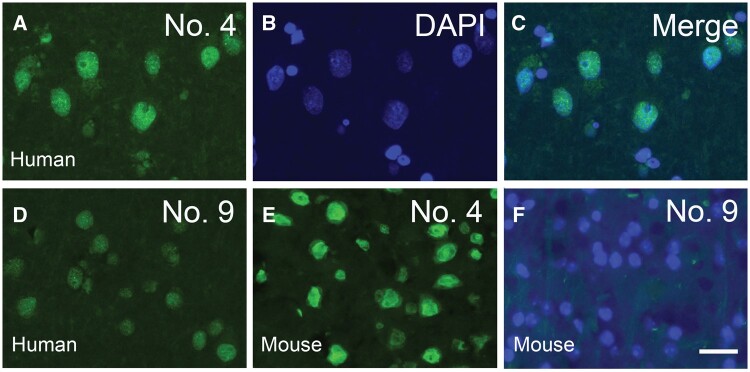
Representative images of immunofluorescence staining using MAbs to visualize normal TDP-43 (green) in the nuclei (blue) of human and mouse cortical tissue. MAb No. 4 **(A–C, E)** serves as an illustration of the 9 MAbs that detect normal nuclear TDP-43 in both the human and mouse cortex. MAb No. 9 is weakly positive in the human brain **(D)**, but completely negative in the mouse brain (**F**). Scale bar, 20 µm.

To assess the cross-reactivity of the MAbs with both human and mouse TDP-43, immunofluorescent staining was conducted on normal human and non-transgenic mouse brain sections (Fig. 3). Nine MAbs exhibited strong positive staining for nuclear TDP-43 in sections of both human and mouse cortex. However, MAb No. 9 did not detect mouse TDP-43 and showed only weak positivity for human nuclear TDP-43.

### Immunoreactivity of phosphorylation-independent MAbs in patient brain tissue

**Figure 4.**
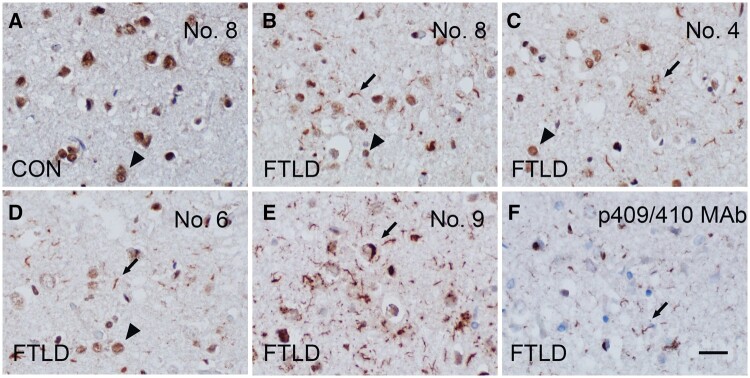
Immunohistochemical features of anti-TDP-43 MAbs in the FTLD-TDP frontal cortex. N-terminal-specific MAb No. 8 reacts with normal nuclear TDP-43 (arrowhead, **A** and **B**) and FTLD cortical TDP-43 pathology (arrow, **B**). CON, normal control cortex. **(C, D)** Representative photos of immunostaining using MAbs that are specific for the RRM1, RRM2, or C-terminal glycine-rich domain show immunoreactivity for both normal nuclear TDP-43 (arrowheads) and pathologic inclusions (arrows). **(E)** MAb No. 9 robustly detects pathological TDP-43 inclusions (arrow), with weak nuclear staining, similar to antibodies specific for p409/410 **(F)**, but MAb No. 9 exhibits a higher extent of TDP-43 pathology overall. Scale bar: 20 µm.

The reactivity of TDP-43 MAbs in brain tissues from FTLD-TDP patients was assessed using immunohistochemical staining on frontal cortical sections (Fig. 4). Semiquantitative analyses for each type of morphologic inclusion (i.e. NCI, DN, NII, and GCI) in FTLD-TDP are summarized in Table 4. MAb No. 8, specific for the N-terminus of TDP-43, exhibited robust reactivity for normal nuclear TDP-43 (Fig. 4A) and detected pathology in the FTLD-TDP frontal cortex (Fig. 4B). The remaining MAbs, specific for the RRM1, RRM2, or C-terminal glycine-rich domain, demonstrated varied immunoreactivity for normal nuclear TDP-43 and weak to strong reactivity for pathologic inclusions, except for MAb No. 9 (Fig. 4E).

**Table 4.** Summary of the immunohistochemical features of the new TDP-43 MAbs in the frontal cortex of FTLD-TDP type a brains.

MAb	Nuclei	NCIs	DNs	NIIs	GCIs
No. 1	−	−	−	−	−
No. 2	+/−	−	−	−	−
No. 3	+++	++	+	+	+
No. 4	+++	++	+	+	+
No. 6	+++	++	+	+	+
No. 8	+++	++	+	+	+
No. 9	+	++	+++	+	+
No. 10	+++	++	+	+	+
No. 11	+++	++	+	+	+
No. 15	+++	++	+	+	+
p409/410	−	++	+	+	+

Nuclei , normal endogenous TDP-43 staining; NCIs, neuronal cytoplasmic inclusions; DNs, dystrophic neurites; NIIs, neuronal intranuclear inclusions: GCIs, white-matter glial cell inclusions; − = none, + = mild, ++ = moderate, or +++ = severe.

### Characterization of MAb No. 9

**Figure 5.**
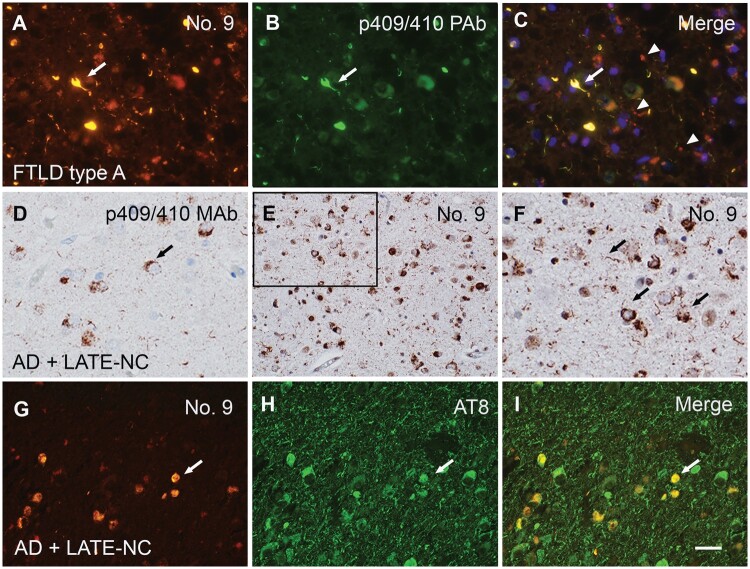
Characterization of MAb No. 9. **(A–C)** Double immunofluorescent staining with MAb No. 9 and the p409/410 PAb of frontal cortex tissue from an FTLD-TDP type A brain. MAb No. 9 detects similar pathologic TDP-43 inclusions as those seen with the p409/410 PAb (arrows) (Pearson’s coefficient value of 0.665), yet reveals additional novel TDP-43 positivity, indicated by fine red dot-like stains (arrowheads) in the merged photo. **(D–F)** MAb No. 9 reveals a more extensive degree of TDP-43 pathology in the amygdala of an AD with LATE-NC brain (AD + LATE-NC), compared to the p409/410 MAb. (F) inset of (E). **(G–I)** MAb No. 9 immunopositivity (cy3, orange) is co-localized (arrows) with AT8-positive tangles (FITC, green) in the medial temporal region of an AD with LATE-NC brain. Scale bars: A–D, F–I, 20 µm; e, 40 µm.

MAb No. 9, targeting the C-terminal region at aa311-360 (Fig. 1), exhibited robust immunoreactivity toward all types of pathological TDP-43 inclusions (i.e. NCIs, DNs, NIIs, and GCIs), with only mild reactivity for normal nuclear TDP-43. The ability of MAb No. 9 to preferentially detect pathologic TDP-43 inclusions is similar to the ability of the p409/410 MAb or PAb, as evidenced by similar staining patterns (Fig. 4). However, MAb No. 9 revealed an overall greater degree of TDP-43 pathology than the p409/410 MAb or PAb (Figs. 4 and 5). Notably, MAb No. 9 could identify more fine neurites in the frontal cortex of FTLD-TDP type A brains (Figs. 4 and 5, Table 4), and more NCIs and more fine neurites in the frontal cortex of FTLD-TDP type B brains (Supplementary Data Fig. S3). However, MAb No. 9 staining was completely colocalized to p409/410 PAb staining in FTLD-TDP type C (Supplementary Data Fig. S3). In the amygdala of AD brains with LATE-NC, MAb No. 9 detected significantly more TDP-43 pathology than the p409/410 MAb (Fig. 5D, E). Double immunofluorescence staining confirmed that MAb No. 9 positivity partially colocalized with AT8-positive tangles, with a Pearson’s coefficient value of 0.712 (Fig. 5G–I).

### Production and characterization of novel phosphorylation-dependent MAb 409/410

Since its initial discovery as the disease protein in FTLD-TDP, abnormal phosphorylation of TDP-43 has been identified as a significant post-translational modification of pathological TDP-43 (1, 10). To gain further insights into TDP-43 phosphorylation, we generated novel phosphorylation-specific MAbs targeting TDP-43-phosphorylated sites, using the phosphorylated 409/410 epitope as a control. Remarkably, we acquired MAbs directed against the p409/410 epitope, uncovering novel pathology that the commercial 409/410 MAb could not detect.

**Figure 6.**
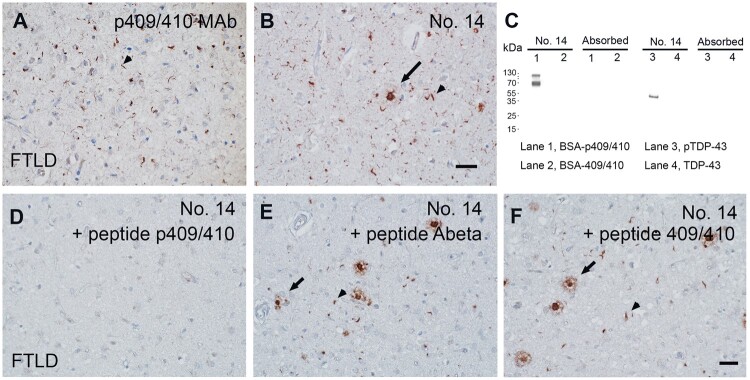
Characterization of MAb No. 14. **(A, B)** MAb No. 14 exhibits pathology in the FTLD cortex akin to that observed with the 409/410 MAb (arrowheads). Additionally, it reveals plaque-like structures (arrow) not seen with the 409/410 MAb. Scale bar: 20 µm. **(C)** The specificity of MAb No. 14 was verified through Western blotting using the BSA-conjugated p409/410 TDP-43 peptide (BSA-p409/410) and full-length recombinant TDP-43 protein phosphorylated in vitro (pTDP-43). Negative controls included BSA-conjugated 409/410 peptide (BSA-409/410) and non-phosphorylated full-length recombinant TDP-43 protein (TDP-43). The immunoreactivity of MAb No. 14 toward the BSA-conjugated p409/410 TDP-43 peptide or phosphorylated full-length TDP-43 was nullified via an absorption test with the p409/410 peptide. **(D–F)** The immunopositivity of MAb No. 14 in the FTLD cortex was abolished through absorption with the p409/410 peptide, while remaining unaffected by the beta-amyloid peptide (Abeta) and non-phosphorylated 409/410 peptide. Arrowheads, FTLD pathology. Arrows, plaque-like MAb No. 14-positive structures. Scale bar: 20 µm.

MAbs were generated against a p409/410-containing TDP-43 peptide conjugated to BSA, OVA, or KLH. A total of 23 MAb clones were obtained through initial ELISA screening against the BSA-conjugated, p409/410-containing TDP-43 peptide. Subsequently, MAb clones negative for p409/410 were excluded using ELISA against the BSA-conjugated, 409/410-containing TDP-43 peptide. The remaining 10 positive MAb clones were further confirmed by immunohistochemistry on brain samples from FTLD-TDP patients. All 10 MAbs were of the IgG1 subclass. As expected, all of these MAbs identified similar pathological TDP-43 inclusions as the commercial p409/410 MAb in FTLD-TDP (Fig. 6). Notably, 4 of these MAbs revealed plaque-like structures in the brain tissue that were undetected by the commercial p409/410 MAb, with No. 14 showing the highest sensitivity. Given the previously unreported ability of the p409/410 MAb to recognize beta amyloid deposits in the brain, the specificity of MAb No. 14 was thoroughly confirmed.

**Figure 7.**
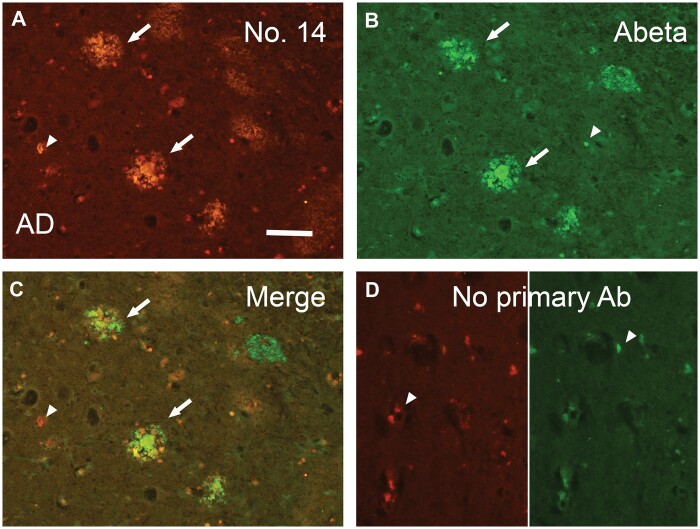
Double immunofluorescence demonstrates partial colocalization of MAb No. 14-positive **(A)** TDP-43 pathology with beta-amyloid (Abeta)-positive **(B)** plaques (arrows) in the frontal cortex of AD brains **(C)**. **(D)** No primary antibody was used as the negative control. Arrowhead, endogenous autofluorescent lipofuscin. Scale bar: 100 µm.

Through immunoblotting, No. 14 reliably identified the p409/410-containing TDP-43 peptide and in vitro phosphorylated recombinant full-length TDP-43. The immunoreactivity of No. 14 in both assays was nullified by absorption with 1 µg/mL p409/410-containing TDP-43 peptide (Fig. 6C). The immunopositivity of MAb No. 14 in FTLD brain tissue was also entirely absorbed by the p409/410 TDP-43 peptide (Fig. 6D). However, the immunopositivity of MAb No. 14 in both FTLD pathology and plaque-like structures persisted after absorption with the beta amyloid peptide (1–42) or TDP-43 409/410 peptide (Fig. 6E, F). Double immunofluorescence staining confirmed that MAb No. 14 positivity partially colocalized with beta amyloid-positive plaques in the AD cortex (Fig. 7), with a Pearson’s coefficient value of 0.769.

**Figure 8.**
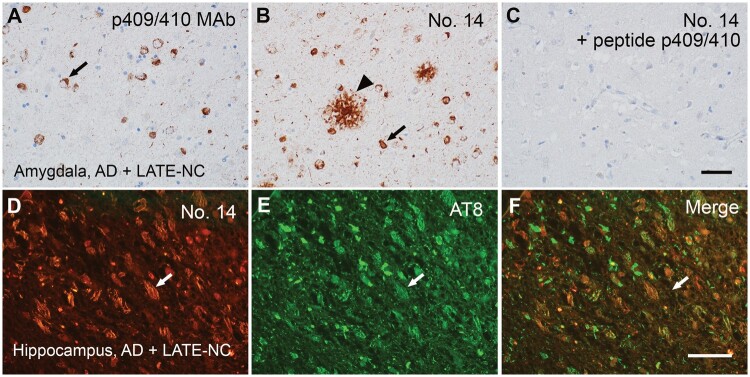
The immunoreactivity of MAb No. 14 in AD with LATE-NC. **(A–C)** MAb No. 14 reveals TDP-43 pathology in the amygdala of an AD brain with LATE-NC, mirroring that revealed by the 409/410 MAb, though No. 14 could also reveal a distinctive plaque-like structure that the 409/410 MAb could not. Notably, all MAb No. 14 immunopositivity was eradicated through absorption with the p409/410 peptide. Scale bar, 20 µm. **(D–F)** Double immunofluorescence demonstrates partial colocalization of MAb No. 14-positive TDP-43 pathology with AT8-positive tangles (arrows) in the hippocampus of an AD brain with LATE-NC. Scale bar: 100 µm.

Furthermore, in the amygdala of an AD brain with LATE-NC, MAb No. 14 revealed pathology similar to that observed with the 409/410 Mab; again, however, No. 14 revealed plaque-like structures not identified with the 409/410 MAb (Fig. 8). The absorption test with the p409/410 peptide abolished both classic and plaque-like TDP-43 pathology, confirming the specificity of MAb No. 14. Double immunofluorescence staining demonstrated partial colocalization of MAb No. 14-positive TDP-43 pathology with AT8-positive tangles (Pearson’s coefficient value of 0.743) in the hippocampus of AD brains with LATE-NC (Fig. 8D–F).

### Biochemical assessment of MAbs

**Figure 9.**
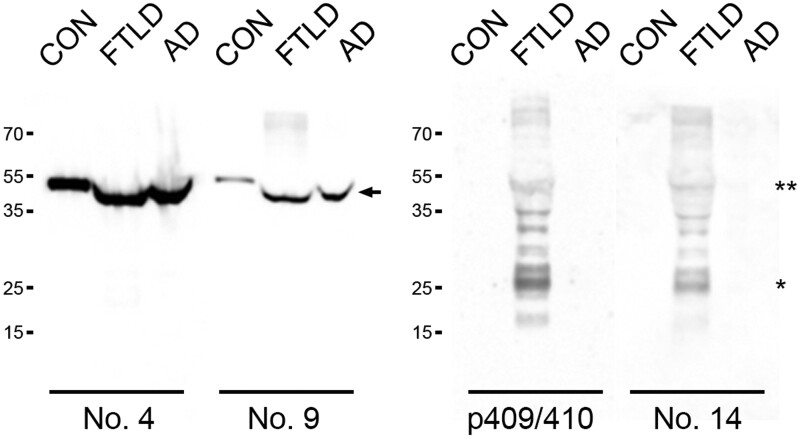
Western blot employing MAbs on human postmortem brain tissue. The sarkosyl-insoluble fraction was obtained from control (CON), FTLD-TDP (FTLD), and AD brains. MAbs No. 4 and No. 9 exhibit strong immunoreactivity with a 43-kDa band (arrow) in control, FTLD-TDP, and AD brain tissue. Similar to the p409/410 MAb, MAb No. 14 reveals abnormal TDP-43 species, including hyperphosphorylated full-length TDP-43 of 45 kDa (**) and higher, as well as C-terminal fragments with bands around 25 kDa (*) in FTLD-TDP brain tissue. Neither the p409/410 nor No. 14 MAbs detect bands in the control and AD brains.

To further confirm the specificity of the MAbs, we performed immunoblot analyses using protein lysates extracted from frozen frontal cortical tissues of FTLD, AD, and normal control brains (Fig. 9). As expected, the phosphorylation-independent MAb No. 4 detected physiological TDP-43, present as a 43-kDa band in FTLD-TDP, AD, and control brains. MAb No. 9 exhibited a similar band pattern, except for a subtle decrease in the relative abundance of physiological TDP-43 in the control brain. Immunoblots using MAb No. 14 revealed the characteristic biochemical profile linked to FTLD-TDP. This profile included the presence of phosphorylated TDP-43 protein at 45 kDa and higher, alongside phosphorylated and C-terminal TDP-43 fragments around 25 kDa. Notably, no band of 43 kDa, corresponding to physiological TDP-43, was detected in the FTLD-TDP brain by MAb No. 14. Moreover, no definite bands were detectable in the AD and control brains. The biochemical signature observed for MAb No. 14 resembles that seen with the p409/410 MAb.

### Establishment of a sandwich ELISA system using MAbs

At present, there is no dependable method for quantifying TDP-43 proteins in biofluids, such as plasma or cerebrospinal fluid (CSF). To assess the potential of the MAbs generated in this study for establishing an assay to detect TDP-43 in CSF or plasma, we evaluated these MAbs using a sandwich ELISA. First, a standard curve for the sandwich ELISA was established using 20 ng/mL of human prokaryotic expressed TDP-43 protein. Second, MAbs No. 3, No. 4, No. 6, and No. 9 were employed to screen for the optimal antibody pair through ELISA. The results revealed that No. 4, when used as the capture antibody, and Biotin-No. 9, as the detection antibody, constituted the most effective antibody pair among the 12 pairs for efficiently detecting TDP-43 in the sandwich ELISA.

**Figure 10.**
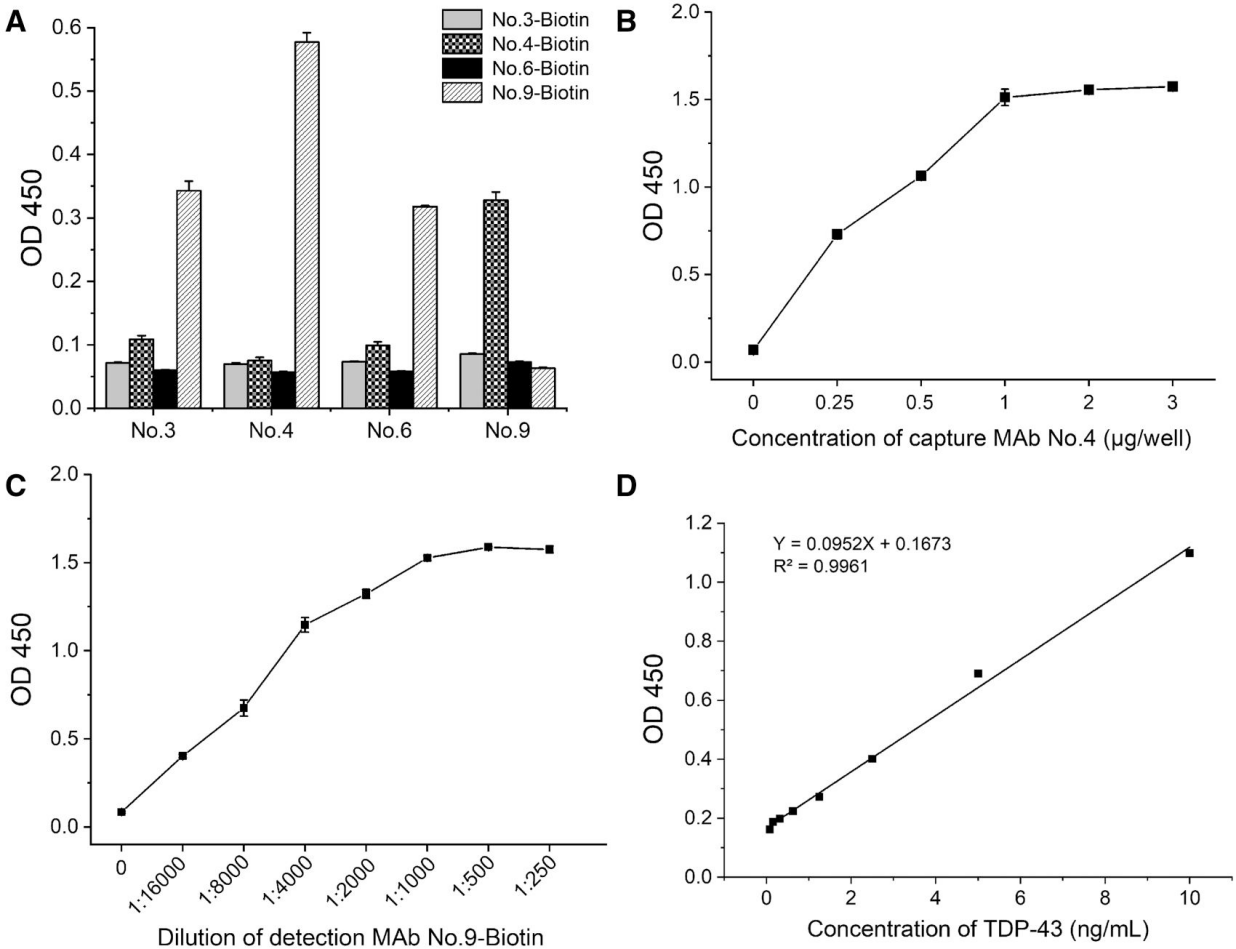
Sandwich ELISA system for detecting TDP-43. **(A)** To screen the antibody pairs using sandwich ELISA analysis, 20 ng/mL of TDP-43 protein was introduced to the plates. The most effective pair was identified based on the highest dilution exhibiting the maximum OD450 values. **(B)** The optimal concentration of the capture antibody, No. 4, was determined through sandwich ELISA across a range of concentrations, from 0.25 to 3 μg/well. **(C)** The optimal dilution of the detection MAb Biotin-No. 9 was determined through sandwich ELISA across a range of dilutions, from 1:250 to 1:16 000. **(D)** Standard curve of optimized sandwich ELISA for detecting TDP-43. Error bars depict the mean ± standard deviation of 3 independent experiments.

To optimize the reaction conditions of the antigen-capture assay, a checkerboard analysis was performed on the serial dilutions of both the capture and detection antibodies. The results indicated that the optimal concentration for immobilizing MAb No. 4 as a capture antibody was 1 µg per well, with optimal performance achieved when the detection MAb No. 9 was diluted to a ratio of 1:1000. The standard curve generated from the optimized sandwich ELISA employing this antibody pair demonstrated a detection limit range from 162.3 to 10 ng/mL (Fig. 10).

## DISCUSSION

In this study, we utilized bacteria-expressed human full-length recombinant TDP-43 protein to produce novel phosphorylation-independent MAbs recognizing epitopes distributed throughout the entire length of TDP-43. Ten of the obtained MAbs were meticulously characterized. Most of these antibodies are valuable for applications, such as immunohistochemistry and Western blotting and they can serve as both capture and detection MAbs in an ELISA. Remarkably, we identified a distinctive MAb, No. 9, which targets an epitope in aa311-360 of the C-terminus. This MAb exhibits preferential reactivity for pathological TDP-43 inclusions, with only mild reactivity for normal nuclear TDP-43. In addition, MAb No. 9 was demonstrated to reveal more pathology in FTLD-TDP type A and type B brains, and AD with LATE-NC brains compared to the p409/410 MAb. Moreover, MAb No. 9 selectively detected human TDP-43, not mouse TDP-43, while the other MAbs demonstrated equal robustness in detecting both human and mouse TDP-43. Furthermore, by utilizing synthetic peptides containing the p409/410 epitope, we developed phosphorylation-dependent MAbs that specifically target the TDP-43 p409/410 epitope. All of the obtained MAbs detected similar pathologic TDP-43 inclusions as the p409/410 MAb. However, remarkably, 4 of these antibodies revealed additional pathology in the LATE-NC brains, with No. 14 demonstrating the highest sensitivity.

Immunostaining with MAbs against TDP-43 on human FTLD-TDP or AD brain tissues can provide insights into the pathophysiology of TDP-43 misfolding and aggregation in human disease. Previous studies have shown that neocortical inclusions in FTLD-TDP are primarily immunopositive for C-terminus-specific MAbs and largely immunonegative for N-terminus-specific MAbs (11, 24). Interestingly, MAb No. 8, an N-terminus-specific MAb generated in this study, showed strong reactivity with brain TDP-43 pathology. Among the 9 phosphorylation-independent TDP-43 MAbs specific for RRM1, RRM2, or the C-terminus, we found a unique MAb, No. 9, which exhibits a heightened affinity for pathological TDP-43 akin to that of the pathology-specific MAb p409/410. In addition, MAb No. 9 was shown to reveal more TDP-43 pathology in FTLD-TDP type A and type B brains, and LATE-NC brains than the p409/410 MAb.

The specificity of the novel MAb No. 9 was thoroughly validated via ELISA, Western blot, immunostaining, and absorption tests. The increased capability of MAb No. 9 to reveal more TDP-43 pathology immunohistochemically in FTLD-TDP type A and type B, and AD with LATE-NC tissues compared to MAb 409/410 may be attributed to the higher affinity of MAb No. 9. However, it is important to note that the higher affinity of MAb No. 9 cannot account for its ability to reveal more fine neurites in the frontal cortex of FTLD-TDP type A brains. There are several potential explanations for the unique immunohistochemistry staining pattern observed with MAb No. 9. First, the pattern could stem from a disease-specific pathological modification to one or more amino acids within the epitope recognized by MAb No. 9. However, this explanation seems unlikely, as all of the novel MAbs were generated using unmodified human recombinant TDP-43. Second, a recent study has proposed that the TDP-43 protein may undergo self-association when interacting with nucleic acids, potentially altering the conformation of TDP-43 or the accessibility of the MAb No. 9 epitope (48). Lastly, it is plausible that under physiological conditions, the epitope specific to MAb No. 9 might be concealed or bound to nucleic acids, making it inaccessible to the antibody in its normal place within the nucleus, but becoming accessible within TDP-43 inclusions. Interestingly, while MAb No. 2 also mapped to a similar region (Fig. 1), it did not display this distinctive immunohistochemical staining pattern shared by MAb No. 9. It is improbable that the 2 MAbs share an identical epitope within this 49 aa-spanning region. Therefore, further epitope mapping is necessary to discern their specific epitopes in this region and to elucidate the discordant nature of their immunoreactivity.

Given the 96% identity between mouse and human TDP-43, it is not uncommon for antibodies developed against human TDP-43 to cross-react with the mouse protein. Notably, MAb No. 9 demonstrates a distinct preference for recognizing human TDP-43, and showing no immunoreactivity with mouse TDP-43. Earlier studies have suggested that antibodies binding to an epitope downstream of aa261 tend to demonstrate specificity for the human variant (24). This propensity is likely attributable to inherent differences in amino acid sequences between human and mouse TDP-43. As a result, this novel TDP-43 MAb, with a notable specificity for human variants, holds significant value for research involving transgenic animal models of TDP-43 proteinopathies. Its utility is particularly crucial in distinguishing between mouse and human TDP-43 C-terminal fragments that accumulate in pathological inclusions.

Notably, MAb No. 9 could detect normal nuclear TDP-43 in SH-SY5Y cells, as determined by immunocytochemistry, although to a lesser degree or intensity than the other MAbs reported in our study (data not shown). Previous findings, demonstrating that TDP-43 could be extracted biochemically with low-stringency buffers from cultured cells (49) but require high-stringency, detergent-containing buffers from central nervous system tissue (1), support a potential structural difference between nuclear TDP-43 in the central nervous system and cultured cells.

MAb 409/410 is commonly employed to investigate and visualize phosphorylated TDP-43 in tissue samples. The antibody’s specificity for phosphorylated TDP-43 makes it a valuable tool in the field of neurobiology and neuropathology, providing insights into the mechanisms underlying neurodegenerative disorders associated with TDP-43 abnormalities. Surprisingly, we obtained MAbs targeting the p409/410 epitope, which revealed additional pathology in AD with LATE-NC brains compared to the commercial 409/410 MAb. Notably, among these antibodies, No. 14 was proven to be the most sensitive. Similar to the commercial 409/410 MAb, MAb No. 14 immunopositivity was colocalized with AT8-positive tangles. However, MAb No. 14 revealed plaque-like TDP-43 deposits that the commercial 409/410 MAb could not detect.

The specificity of MAb No. 14 was validated through ELISA, Western blot analysis using peptides containing p409/410, immunostaining, and absorption tests. In light of the plaque-like TDP-43 immunopositivity revealed by MAb No. 14, further confirmation of No. 14’s specificity involved absorption tests on patient brain sections with the beta-amyloid peptide, in addition to the peptide containing the p409/410 epitope. The specificity of MAb No. 14 was further supported by the fact that the No. 14-positive deposits in the plaques were only found in particular regions, such as the frontal cortex and hippocampus; No. 14 was only partially co-localized with beta-amyloid.

The enhanced capability of MAb No. 14 to reveal more TDP-43 pathology in AD with LATE-NC compared to the p409/410 MAb may be attributed to the higher affinity of MAb No. 14. Additionally, it could be due to the possibility that TDP-43 protein in the vicinity of the p409/410 epitope may have different conformations and that our No. 14 MAb could recognize some conformations better than MAb 409/410. The identification of TDP-43-immunopositive deposits with amyloid plaques will have significant implications for understanding the pathogenesis of AD pathology and potential treatment strategies. Interestingly, similar to the p409/410 MAb, MAb No. 14 failed to detect bands in frozen AD brain tissue via immunoblotting. This discordance between the immunoblotting and immunostaining results could be attributed to the low level of No. 14-positive TDP-43 protein in the tissue or the use of a suboptimal tissue extraction method. Further protein chemical study is needed to interpret the discordance.

Evidence for the biochemical heterogeneity of aggregated TDP-43 has been reported previously (25, 50, 51). Laferrière et al showed that the molecular properties of TDP-43 inclusions correlate with specific neuropathological subtypes (25). While it remains to be seen whether these biochemical differences indeed represent strains or species with specific cell tropism and toxicity, several emerging reports support this view: distinct seeding activities and toxicity of TDP-43 aggregates have been described both in vitro and in vivo (25, 52, 53). We further postulate that TDP-43 species might serve as a link between a defined TDP-43 pathology and a specific clinical phenotype. Antibodies targeting the new toxic TDP-43 species could potentially establish a connection between pathology and clinical phenotype (54). In future work, we will further characterize MAbs No. 9 and No. 14 through a comprehensive neuropathological survey using brains affected by AD with LATE-NC.

### Conclusion

The novel MAbs reported here will be powerful tools for further dissecting the biochemical basis of FTLD-TDP and AD with LATE-NC cases. They may become essential for preclinical evaluations of novel therapeutics targeting TDP-43 aggregation and may also find application as potential immunotherapies. There is a pressing demand for FTLD-TDP-specific biomarkers (55), and ELISAs capable of accurately measuring TDP-43 levels in CSF and/or plasma are crucial not only for diagnosis but also for monitoring responses to disease-modifying therapies in FTLD-TDP and other TDP-43 proteinopathies (56). Consequently, we anticipate that the set of novel MAbs outlined in this study will serve as valuable tools for future patient-oriented and experimental studies of TDP-43 proteinopathies.

## Supplementary Material

nlae042_Supplementary_Data

## Data Availability

All data generated or analyzed during this study are included in this published article. The antibodies will be shared with interested researchers upon request and will be made commercially available.
