# Supplementary figures and images for: Production and characterization of novel monoclonal antibodies against pathological human TDP-43 proteins
Source: J Neuropathol Exp Neurol. 2024 May 10;83(8):655–69. doi: 10.1093/jnen/nlae042 (PMC11258413; doi:10.1093/jnen/nlae042)

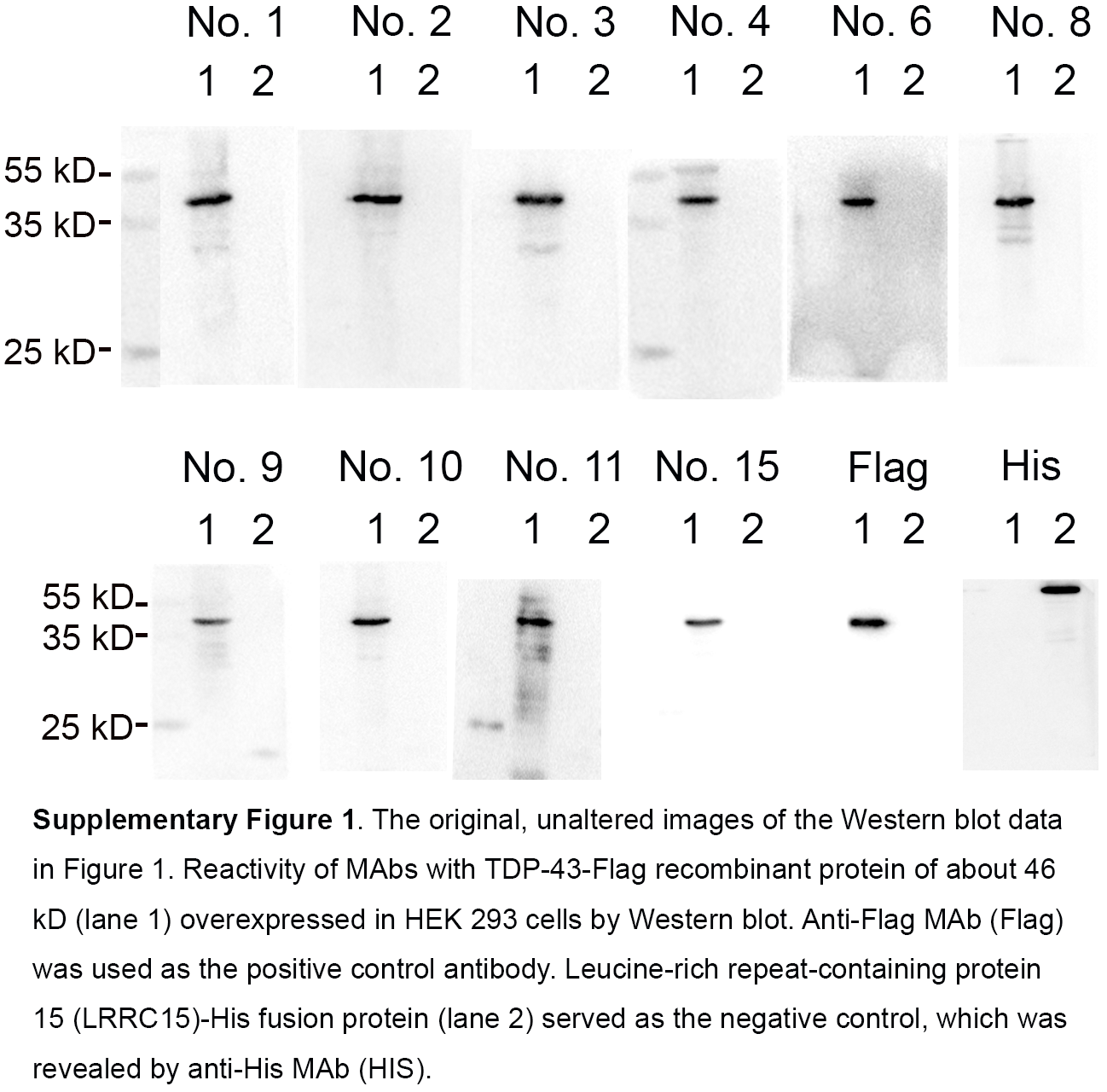

Supplement: nlae042_Supplementary_Data [file nlae042_supplementary_data.zip › nlae042_Supplementary_Data/Supple Fig 1-1.tif]

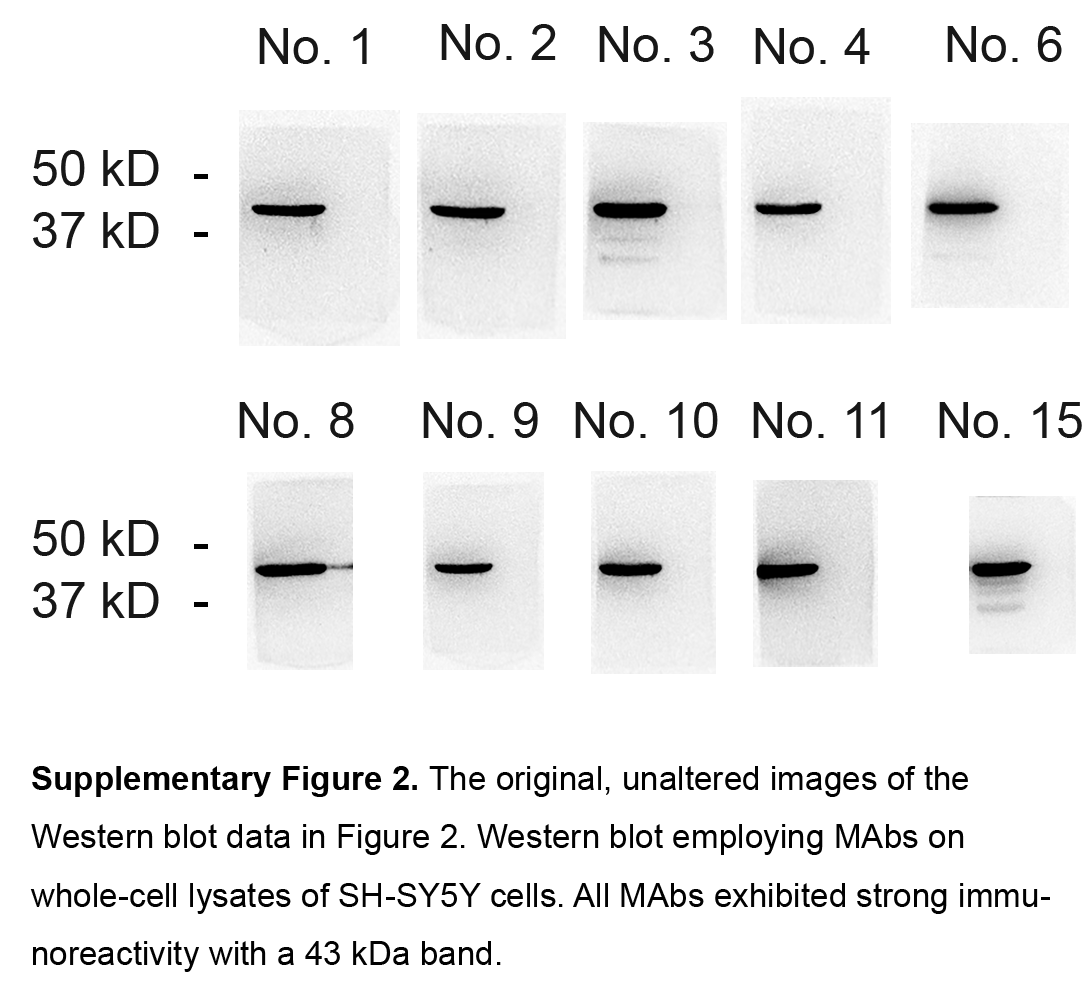

Supplement: nlae042_Supplementary_Data [file nlae042_supplementary_data.zip › nlae042_Supplementary_Data/Supple Fig 2.tif]

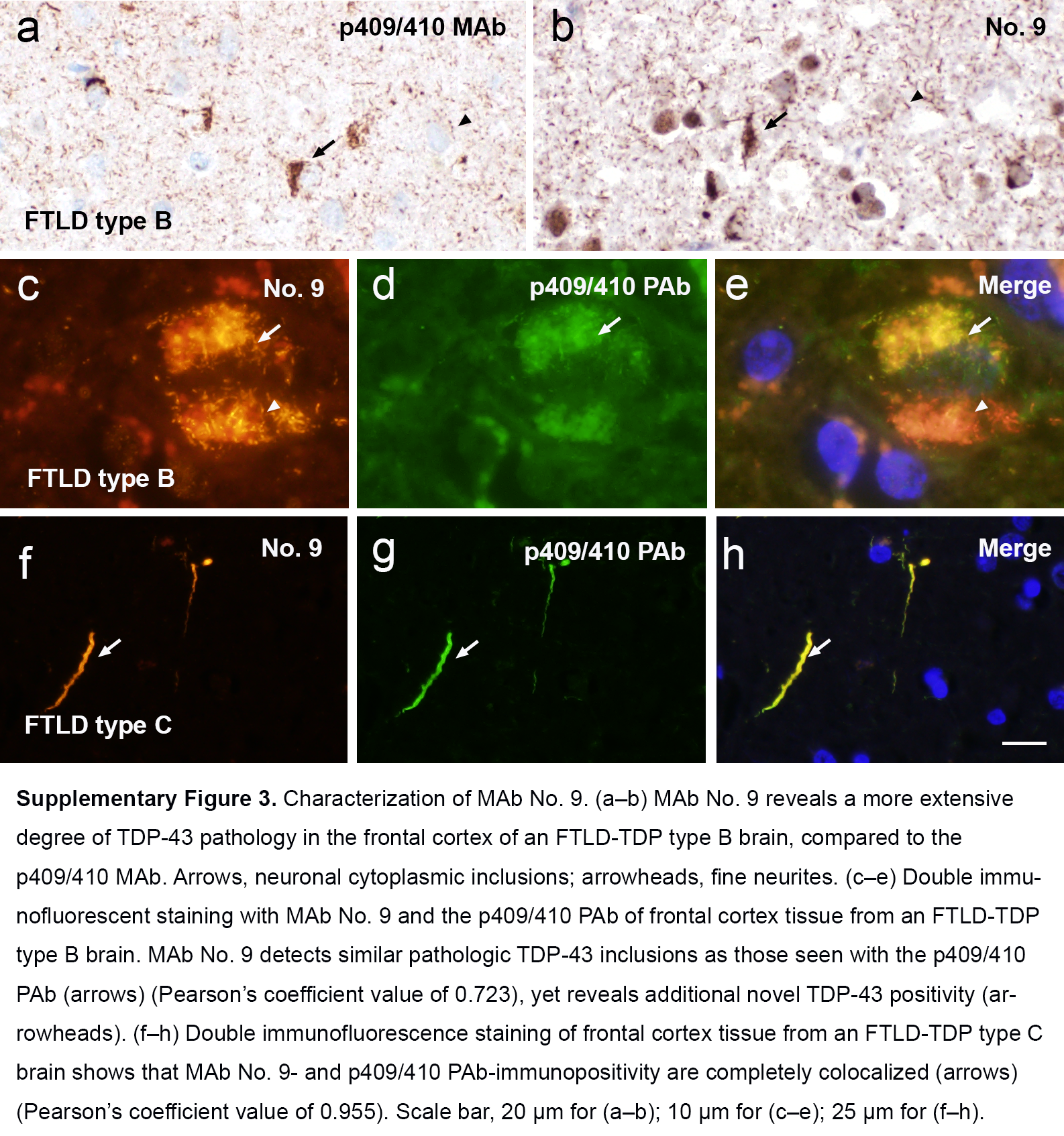

Supplement: nlae042_Supplementary_Data [file nlae042_supplementary_data.zip › nlae042_Supplementary_Data/Supple Fig 3.tif]
